# Supplementary material for: Does low fertility indicate better reproductive health status? Evidence from nationally representative survey in India
Source: PLoS One. 2025 Aug 12;20(8):e0329375. doi: 10.1371/journal.pone.0329375 (PMC12342277; doi:10.1371/journal.pone.0329375)
Supplement: S1 Table — (DOCX) [file pone.0329375.s001.docx]

**S1 Table. Sensitivity analysis using Poisson regression models based on alternate Reproductive Health Index (RHI) specifications.**

| Variables | RHI1 Coef. [95% CI] | RHI2 Coef. [95% CI] | RHI3 Coef. [95% CI] | RHI4 Coef. [95% CI] |
| --- | --- | --- | --- | --- |
| CEB |  |  |  |  |
| 1(®) |  |  |  |  |
| 2 | 0.00[-0.00,0.01] | -0.01***[-0.02, -0.01] | 0.00[-0.01,0.01] | 0.01[-0.00,0.01] |
| 3 | 0.00[-0.00,0.01] | -0.03***[-0.04, -0.03] | 0.00[-0.00,0.01] | 0.01**[0.01,0.02] |
| ≥ 4 | -0.02***[-0.03, -0.01] | -0.07***[-0.09, -0.06] | -0.02***[-0.03, -0.01] | 0.00[-0.01,0.01] |

**Note:** Values represent Poisson regression coefficients with 95% confidence intervals. All models control for age, age at marriage, age at first birth, social group, religion, mass media exposure, wealth quintile, region, and place of residence.

**RHI1** (Main index): Includes all seven indicators — BMI status, Anaemia status, Low Birth Weight, Antenatal care by skilled provider, Delivery assistance by skilled provider, Stillbirth or miscarriage, and Caesarean delivery.

**RHI2:** Excludes Caesarean delivery.

**RHI3:** Excludes Stillbirth or miscarriage.

**RHI4**: Excludes Antenatal care and BMI status.
