# Supplementary material for: Does low fertility indicate better reproductive health status? Evidence from nationally representative survey in India
Source: PLoS One. 2025 Aug 12;20(8):e0329375. doi: 10.1371/journal.pone.0329375 (PMC12342277; doi:10.1371/journal.pone.0329375)
Supplement: Attachment — Submitted filename: Comments.docx [file pone.0329375.s002.docx]

The work is exciting and holds significant future implications. The authors have provided a detailed structure with well-articulated sections, including the Introduction, Results, Discussion, and Conclusion. However, I have a few minor observations for improvement:

Abstract Section

1. **Abstract Section**:

In the Results section of the abstract, I recommend starting with the variation in the RHI score by fertility level and how this score changes by including socio-economic factors in the regression. This could be followed by describing the RHI score across various socio-economic demographic groups and geographical regions. This sequence may help present the results more effectively per the stated objectives.

2. **Variables and Measurement, and Statistical Analysis Section**:

- The section uses the phrase "women’s holistic well-being" repetitively. For example, it appears five times between lines 205 and 213. Rephrasing some of these instances might be more concise and effective to avoid redundancy.
- The sentence, “Each indicator is associated with a specific threshold value, beyond which a woman is considered an RHI score,” in line 186, may be removed or further clarified if it holds significant value. If there is an essential explanation behind this sentence, please provide more details for clarity.
- The lines from 203 to 206: "Furthermore, we use a composite index to identify the principal contributors of RHI. This approach provides a comprehensive understanding of the critical determinants influencing women's holistic well-being in the studied population," appears to repeat information already presented in the Variables and Measurement section. Consider removing this for conciseness.

3. **Discussion Section**:

- The term "these burdens" in line 332, requires further explanation. Please provide additional context for what specific burdens are being referred to in this section.
- It would be important to include a discussion of how low fertility may impact reproductive health. A brief explanation of how lower fertility rates contribute to reproductive health outcomes would add valuable insight.
- Additionally, I encourage the authors to incorporate the theoretical components introduced in the Introduction section into the Discussion. This integration could effectively connect the study's framework and enrich the analysis of the results.

Overall, these suggestions are meant to enhance clarity and coherence while maintaining the strong quality of the work.
