# Supplementary material for: Does low fertility indicate better reproductive health status? Evidence from nationally representative survey in India
Source: PLoS One. 2025 Aug 12;20(8):e0329375. doi: 10.1371/journal.pone.0329375 (PMC12342277; doi:10.1371/journal.pone.0329375)
Supplement: Attachment — Submitted filename: plosone_review.docx [file pone.0329375.s003.docx]

**Review**

**Does Low Fertility Indicate Better Reproductive Health Status? Evidence from Nationally Representative Survey in India**

**Manuscript Number:** **PONE-D-25-01359**

This cross-sectional study attempts to explore the association between declining fertility rates and women's reproductive health in India, considering socioeconomic and demographic factors as well as regional variations. The objective of this paper is quite relevant & interesting, but has some shortcomings which needs major revisions & rework. **Hence, upon preliminary review, my recommendation is for a major revision before considering the manuscript for publication.**

**Followings are some of my observations that needs further clarification and revision:**

1. The article requires substantial revision to address grammatical problems and repetitive sentences (e.g., lines 355-359). Furthermore, the article appears to be very lengthy, and the assertions in the findings, discussions, and conclusion sections are very repeated.
2. Line no. 106-107: ‘By integrating these …….low fertility on holistic well-being”. I have a serious concern in using the term ‘holistic well-being of women’ which can include both mental and social well-being as well (as the author mentioned in 99-101). But in the analysis, there are no indicators on social or mental well being of women. As a result, it is better to avoid using this term or to explain how your seven indicators can justify mental and social well-being.
3. Line no. 224-25: ‘However, 9.78% of deliveries ……remains insufficient.’ Lower percentages of deliveries by skilled persons does not always imply lower access to health care facilities. What about the families who don’t opt for such deliveries and prefer delivery at home?
4. Line no. 258-259: ‘Regarding regional variations…..RHI’. Even northern regions have highest mean RHI (4.42). But the author doesn’t mention that. Additionally, interpretation of regression mentioned (in line no. 316-318) north-eastern region showing lower likelihood of RHI, while the values are positive. Please rework and rewrite the section.
5. In some cases, the OR values are very low. Plus, the author needs to mention the high or low RHI score range as the scores does not vary much (such as 4.33 in West and 4.35 in South). Please elaborate on how far it is justified to say that regional disparities exist (line 316-318)?
6. Line 318-319: ‘Additionally, residence plays…. counterparts (Model 3)’. In model 3 Rural and urban odds are not significant. Therefore, no difference between rural and urban areas.
7. Line 297-300: ‘Model 3 highlights….wealth index’. Please elaborate, as the models do not show that.
8. Please recheck the Poisson regression model. Odds ratio can’t be negative. An odds ratio greater than 1 indicates an increased risk associated with the exposure, while an odds ratio less than 1 indicates a decreased risk. Do the values in Table 4 represent odds or log odds? If log odds, then the interpretation should alter.
9. The author tries to investigate the spatial variation. What about the role of clustering effect on depended variable due to hierarchical nature of NFHS data? Can Poisson regression model consider that effect?
