# Supplementary material for: Does low fertility indicate better reproductive health status? Evidence from nationally representative survey in India
Source: PLoS One. 2025 Aug 12;20(8):e0329375. doi: 10.1371/journal.pone.0329375 (PMC12342277; doi:10.1371/journal.pone.0329375)
Supplement: Attachment — Submitted filename: Response to Reviewers.docx [file pone.0329375.s004.docx]

Reviewer #1: The paper has novelty and significance in respect to current women health condition in India. But this paper has been analysed and written very well but it has some structural problem that need to improve for better readability of the paper. Here are my following suggestions.

Reply: We are sincerely grateful for the time and efforts for the reviews of our manuscript, “Does Low Fertility Indicate Better Reproductive Health Status? Evidence from Nationally Representative Survey in India.” We appreciate the constructive feedback, which has helped us to strengthen the manuscript significantly.

Comment 1. Line 45: The authors have written (coefficient = -0.05; CI: -0.06, -0.05), what is the relevance of these values in abstract, I would suggest to remove the values from abstract and only focus on the outcome of analysis.

Reply: Thank you. We have removed the coefficient values and retained only the outcomes in the abstract.

Comment 2. I do not feel the necessary of separate Introduction and Fertility Transition and Reproductive Health section, rather I would suggest to integrate them and strengthen the background of the study.

Reply: We appreciate this suggestion. These sections have been merged and the background has been strengthened (see lines 97–120 and 137–139).

Comment 3. Do you really need this sentence in line 162-163- “The International Institute for Population Sciences (IIPS) in Mumbai conducted the survey with funding from the Government of India's Ministry of Health and Family Welfare”. I don’t feel so. Similar for line 166-167. Streamline the line 161-168, and remove unnecessary lines.

Reply: Thank you for this suggestion. We addressed this comment and removed all the lines from the data as suggested.

Comment 4. After reading the whole methodology I feel that it needs to rearrange, as my previous comment, remove unnecessary writing for data, sample size and Variables and their measurement section. Combine them together in one section, and named the section- ‘study variables, measurements and sources’ something like that. Then in that section first paragraph write data source and samples, then onwards variable measurements.

Reply: Addressed. We have created a two section titled Data Sources and Study Variables and their Measurements, combining the data, variables, and source sections.

Comment 5. Line 215: Do you want to cite STATA, if not then what is the need for writing this-(Stata Corporation, College Station, TX), as you already stated that you used STATA 17.

Reply: Thank you for this suggestion. The citation has been removed.

Comment 6. I would Like to give citation or equation in like 202-206

Reply: We have added the composite index equation and appropriate citations (see lines 180–183).

Comment 7. I would like to give regression equation forms of two poison regressions.

Reply: Thank you for this suggestion. We have addressed the comment and added a regression equation in the methodology section (lines 186-191).

Comment 8. Line 2015-2017- you wrote that “We use ArcMap for creating maps. The shape file for the maps is downloaded from the official Demographic Health Survey (https://dhsprogram.com/data/available-datasets.cfm) website”. If you really feel that you need to mentioned this, concise the sentences.

Reply: Thank you for the suggestion. We have revised the sentence for clarity and conciseness. It now reads: “The shapefile used to generate the map is freely available from the official Demographic and Health Survey (DHS) website: https://dhsprogram.com/data/available-datasets.cfm. The map is prepared by the author.”

Comment 9. In table 1: what is the meaning of given two column for category ? instead you simple in one column, write category and in bracket yes/no (category (yes/no)), is not it represent the same as you did. Think. What you did makes the paper structure unesthetic and reader will lose the concentration while reading.

Reply: Thank you for your valuable feedback. We acknowledge that having two separate columns for categories may affect the readability of the table. Based on your suggestion, we have reformatted Table 1 with a single column for each variable with values (Yes/No) to improve readability and aesthetics.

Comment 10. Table 2: similar problem like table one, you can simple write total value and % in bracket- 20,213 (9.8).

Reply: Thank you for your comment. We have deleted the second percentage column and merged the total values and percentages into a single column (e.g., 20,213 (9.8%)) to improve readability and ensure consistency across all categories.

Comment 11. I do not understand the meaning of “No one” in table 2, can you clarify and reframe.

Reply: We have replaced “No one” with “Unskilled provider/No assistance” to clarify that this category includes cases where women either received assistance from an unskilled provider or had no assistance.

Comment 12. In table 2. For Ever experienced Still Birth/miscarriage- you have written separate percentage for yes and for no, together 100%. Then what is the meaning of given both yes and no, if 74.56 % is for yes, then rest of the % will by default become no. can you explain?

Reply: We appreciate this comment. We have removed the “No” row and retained only the 'Yes' category. A footnote has been added for clarity.

Comment 13. In line 246- you write a heading of a section “Mean number of RHI in background characteristics-wise”, I won’t do that, mean is the analysis you did, it does not represent the content of the section. Rather write “socio-economic/ecological/political/cultural background of RHI”

Reply: We have removed the line Mean number of RHI and addressed this comment by adding the ‘Socio-economic background of RHI’.

Comment 14. Which one is figure 1?, author should have basic responsibility to frame the paper.

Reply: Thank you for your suggestion. Figures are provided separately in TIFF format as per journal submission guidelines.

Comment 15. I don’t feel the necessity of writing composite score in the title “District-level Variation in Women’s Reproductive Health Composite Score”

Reply: The title has been revised as suggested.

Comment 16. Where is figure 2?

Reply: The figure is provided separately in the TIFF file as per the journal’s guidelines.

Comment 17. Table 4, what the value inside table represents? standard error? or coefficient? or something else. How do I know? This reflects the lack of professionalism by the authors. I would suggest to follow good article, and carefully check how they represent the regression table in the paper.

Reply: Thank you for this suggestion. The table now included coefficients and confidence intervals.

Comment 18. Your policy suggestions are too generals, based on your study, What policy measure will you take if you become the sole responsible authority.

Reply: The policy implication section has been revised to reflect precise and actionable recommendations derived from the findings of the study.

Comment 19. Overall, I feel paper is lengthy, and some part of the writing is exaggerated and over romanticised.

Reply: Thank you. We have revised the introduction and reduced redundancy to improve conciseness.

Over all paper is interesting, I feel this structural change make the paper more concise and increase the understandability and readability of the non-subject readers also. I recommend the paper for above minor, not major revision.

Reply: Thank you. The manuscript has been revised accordingly for enhanced readability and organisation.

Reviewer #2: The manuscript presents a rigorously conducted study characterised by a high level of technical competence. The data is robust, and the conclusions drawn are substantiated by the experiments and analyses executed. The statistical analysis is both appropriate and comprehensive, which enhances the reliability of the findings and their interpretation. Furthermore, the authors have made all underlying data available in accordance with PLOS Data policies, thereby ensuring transparency. The manuscript is well-structured and articulately written in standard English, rendering the content both accessible and easily comprehensible.

Reply: Thank you for your supportive feedback. We appreciate your positive evaluation.

Reviewer #3: This cross-sectional study attempts to explore the association between declining fertility rates and women's reproductive health in India, considering socioeconomic and demographic factors as well as regional variations. The objective of this paper is quite relevant & interesting, but has some shortcomings which needs major revisions & rework. Hence, upon preliminary review, my recommendation is for a major revision before considering the manuscript for publication.

Reply: We sincerely thank the reviewer for acknowledging the relevance and importance of our study. We have carefully addressed all suggested revisions to improve the clarity, structure, and interpretation. We hope the revised manuscript meets the expectations for further consideration.

Followings are some of my observations that needs further clarification and revision:

Comment 1. The article requires substantial revision to address grammatical problems and repetitive sentences (e.g., lines 355-359). Furthermore, the article appears to be very lengthy, and the assertions in the findings, discussions, and conclusion sections are very repeated.

Reply: Thank you for your observation. We have thoroughly revised the manuscript to correct grammatical errors and improve clarity. We have deleted the repetitive sentences from the findings and discussion.

Comment 2. Line no. 106-107: ‘By integrating these …….low fertility on holistic well-being”. I have a serious concern in using the term ‘holistic well-being of women’ which can include both mental and social well-being as well (as the author mentioned in 99-101). But in the analysis, there are no indicators on social or mental well being of women. As a result, it is better to avoid using this term or to explain how your seven indicators can justify mental and social well-being.

Reply: We agree with the concern. The phrase “holistic well-being” has been replaced with more precise terms, such as “reproductive health,” in relevant sections to maintain objectivity.

Comment 3. Line no. 224-25: ‘However, 9.78% of deliveries ……remains insufficient.’ Lower percentages of deliveries by skilled persons does not always imply lower access to health care facilities. What about the families who don’t opt for such deliveries and prefer delivery at home?

Reply: Thank you for this important observation. We agree that lower rates of skilled delivery attendance may not always reflect limited access to healthcare services. Cultural preferences, traditional beliefs, and personal choices can also influence the decision to deliver at home without skilled assistance. To address this, we have revised the statement to acknowledge that while the proportion of unskilled deliveries may indicate service gaps, it may also reflect the voluntary preference for home births in certain communities.

Comment 4. Line no. 258-259: ‘Regarding regional variations…..RHI’. Even northern regions have highest mean RHI (4.42). But the author doesn’t mention that. Additionally, interpretation of regression mentioned (in line no. 316-318) north-eastern region showing lower likelihood of RHI, while the values are positive. Please rework and rewrite the section.

Reply: Thank you for the comment. You are correct that the Northern region shows the highest mean RHI score in the descriptive analysis. However, the difference between the North and North Eastern regions becomes statistically insignificant in the regression after adjusting for covariates. Therefore, we rely on the descriptive means for interpretation in this case. Also, we rewrite the section mentioned above.

Comment 5. In some cases, the OR values are very low. Plus, the author needs to mention the high or low RHI score range as the scores does not vary much (such as 4.33 in West and 4.35 in South). Please elaborate on how far it is justified to say that regional disparities exist (line 316-318)?

Reply: Thank you for pointing this out. We have corrected the earlier error where coefficients were mistakenly referred to as odds ratios. These are now properly labeled as Poisson regression coefficients. As for the regional variation, we have included the actual RHI score range (4.09 to 4.42) in the results for clarity. Since the difference is slight and the North Eastern region’s coefficient is not statistically significant, to avoid overstatement, we removed the line referencing regional disparities.

Comment 6. Line 318-319: ‘Additionally, residence plays…. counterparts (Model 3)’. In model 3 Rural and urban odds are not significant. Therefore, no difference between rural and urban areas.

Reply: Thank you for pointing this out. We agree that the difference between rural and urban residence is not statistically significant in Model 3. We have revised the sentence in the results section.

Comment 7. Line 297-300: ‘Model 3 highlights….wealth index’. Please elaborate, as the models do not show that.

Reply: Thank you for this helpful observation. We agree that the original interpretation overstated the role of mass media and wealth index in mitigating the effects of higher parity. We have revised the sentence to reflect the results of Model 3 more accurately.

Comment 8. Please recheck the Poisson regression model. Odds ratio can’t be negative. An odds ratio greater than 1 indicates an increased risk associated with the exposure, while an odds ratio less than 1 indicates a decreased risk. Do the values in Table 4 represent odds or log odds? If log odds, then the interpretation should alter.

Reply: Thank you for pointing this out. You are absolutely correct; odds ratios cannot be negative. In the original manuscript, we mistakenly referred to Poisson regression coefficients as odds ratios (OR), which was inaccurate. The values presented in Table 4 are actually log-linear coefficients, not odds ratios. We have corrected the terminology throughout the results and discussion sections. All interpretations have been revised accordingly to reflect the direction and magnitude of association in terms of coefficients, not ORs.

Comment 9. The author tries to investigate the spatial variation. What about the role of clustering effect on depended variable due to hierarchical nature of NFHS data? Can Poisson regression model consider that effect?

Reply: Thank you for your comment. It is also true that NFHS has used multistage stratified clustering sampling; it already adjusted the hierarchical nature of the dataset using the design effect. Therefore, I believe that the use of Poisson regression in this dataset is suitable.

Reviewer #4: 1. The title of the paper does not reflect the analysis presented; the author does not analyse low fertility but rather focuses on the factors associated with women’s reproductive health.

Reply: Thank you for your feedback. We agree that the analysis primarily focuses on women's reproductive health, but this is intentionally framed within the broader context of India's ongoing fertility transition. The study aims to explore how reproductive health outcomes are shaped during a period of declining fertility, which justifies the use of “low fertility” in the title. We believe the current title captures the thematic intent of the paper and its relevance to the demographic shift under study.

Comment 2. The abstract of the paper does not follow the journal's format.

Reply: Thank you for your observation. We have revised the abstract into different sections, such as the introduction, methods, results, and conclusion.

Comment 3. The introduction section does not clearly mention the research gap and the objective of the paper.

Reply: Thank you for your comment. We have revised the introduction to clearly state the research gap and objectives of the paper. This has been addressed in lines 125-132 of the revised manuscript.

Comment 4. The concept of the Reproductive Health Index (RHI) is not clearly defined in the introduction. The benefits and how NFHS data can be used to measure this index should be explained more clearly.

Reply: Thank you for this helpful comment. We have now clarified the concept of the Reproductive Health Index (RHI) in the introduction (128-130). A more detailed explanation of the selected NFHS indicators and how the index is constructed is provided in the methodology section.

Comment 5. The limitations of the paper should be included in the discussion section, not the introduction section.

Reply: Thank you for this suggestion. We have removed the limitations from the introduction part and addressed them after the discussion section, as recommended.

Comment 6. The justification for using the Poisson regression model should be clarified, especially over other models, such as the negative binomial regression.

Reply: Thank you for this thoughtful observation. You are correct that negative binomial regression is often a preferred alternative for count data when overdispersion is present. However, in our study, we retained the Poisson regression model as the variance and mean of the outcome variable were relatively close. To confirm its suitability, we conducted a Pearson goodness-of-fit test, which indicated a good model fit with no evidence of overdispersion (χ² = 45,143.74; df = 165,254; p = 1.000). Therefore, we proceeded with the Poisson model in our final analysis.

Comment 7. The justification for the equal weighting of indicators in the RHI should be explained. The author could conduct a sensitivity analysis to support this approach.

Reply: Thank you for your valuable suggestion. To assess the robustness of our composite Reproductive Health Index (RHI), we conducted a sensitivity analysis by systematically altering the index composition; we are removing one indicator at a time—and re-estimating the Poisson regression models. The results across all four variations (RHI1 to RHI4) showed consistent direction and statistical significance in key associations, particularly for the number of children ever born (CEB). This consistency supports the reliability of the equal weighting scheme and the stability of our findings. A summary of this sensitivity analysis has been included in the appendix (see S1 Table).

Comment 8. The Northeastern region is associated with higher RHI scores despite socioeconomic disadvantages; this finding needs more discussion.

Reply: Thank you for your insightful comment. We agree that this finding is important and have expanded the discussion to reflect on potential contextual factors—such as cultural health practices, community-level health initiatives, and better maternal service utilisation in parts of the Northeastern region that may contribute to higher RHI scores despite overall socioeconomic disadvantage.
