# Supplementary material for: Does low fertility indicate better reproductive health status? Evidence from nationally representative survey in India
Source: PLoS One. 2025 Aug 12;20(8):e0329375. doi: 10.1371/journal.pone.0329375 (PMC12342277; doi:10.1371/journal.pone.0329375)
Supplement: Attachment — Submitted filename: Response_to_Reviewers_auresp_2.docx [file pone.0329375.s005.docx]

Reviewer #3: The article addresses a crucial aspect of women's reproductive health and its association with low fertility rates in India. This topic remains relatively underexplored, making the study particularly relevant in the Indian context. After an initial round of revisions, the manuscript is now well-organized and clearly written. The following are some minor suggestions that may further improve clarity:

Reply: Thank you very much for your encouraging feedback and for acknowledging the relevance and clarity of our revised manuscript. We sincerely appreciate your constructive suggestions and have carefully considered each of them to further improve the clarity and overall quality of the paper.

Comment 1. Kindly assess the level of multicollinearity—particularly among variables such as age, age at marriage, and age at first birth—prior to conducting the regression analysis, and include the findings in the manuscript.

Reply: Thank you for your valuable suggestion. We have assessed multicollinearity among the key age-related variables (age, age at marriage, and age at first birth) using the Variance Inflation Factor (VIF). All values were below 5 (VIF range: 1.02–3.54), indicating no major multicollinearity concern. This information has been included in the revised methodology section.

Comment 2. Please examine the potential presence of endogeneity, as certain variables used in constructing the Reproductive Health Index (e.g., history of stillbirth/miscarriage, type of delivery assistance) may influence the number of children ever born (CEB). A rationale for the absence of endogeneity in the analysis should be provided.

Reply: Thank you for this valuable comment. We agree that certain indicators, such as history of miscarriage or type of delivery assistance, may conceptually influence or be influenced by fertility behaviours. To address this, we conducted a sensitivity analysis using alternate specifications of the Reproductive Health Index (RHI), including versions that exclude these potentially endogenous components. The results remained consistent in direction and significance, suggesting that any endogeneity is unlikely to meaningfully bias the findings. As this assessment is already reflected in our supplementary analysis (see S1 Table), we have not added a separate discussion in the manuscript.

Comment 3. Specify the states included in each of the six regional categories, or cite the source from which the regional classification was derived.

Reply: Thank you for this suggestion. We have now specified the states included in each of the six regional categories in the Methodology section of the revised manuscript.

Comment 4. In the methodology section, please justify the use of the Poisson regression model. Also, check for the presence of overdispersion or under dispersion in the data and report the findings.

Reply: Thank you for the suggestion. While the rationale for using the Poisson model was already included in the methodology section, we have now tested for overdispersion using the Pearson chi-square goodness-of-fit test. The dispersion statistic was close to 1 (χ² = 45,143.74, p = 1.000), indicating that the Poisson model is well-suited. This clarification has been added to the revised manuscript.

Comment 5. It is recommended to present Relative Risk Ratios (RRR) in Poisson regression results instead of regression coefficients for clearer interpretation of the results.

Reply: Thank you for this suggestion. We have now updated Table 4 and the associated text to report exponentiated Poisson regression results, presenting Incidence Rate Ratios (IRRs) for clearer interpretation. This change enhances the readability and understanding of the model outputs.

Comment 6. Ensure that all figures have appropriate titles and are properly numbered.

Reply: Thank you for your suggestion. We have addressed this comment by ensuring that all figures are now properly numbered Fig 1 and Fig 2 and consistently cited in the manuscript.

Reviewer #4: 1. The introduction section includes limited literature; the authors need to add more recent studies.

Reply: Thank you for your valuable suggestion. We have now revised the Introduction section to include more literature.

Comment 2. The authors should provide more information about the NFHS data, including the questions asked, which questions were used in this study, and the topics covered by NFHS.

Reply: Thank you for this valuable comment. In response, we have elaborated on the NFHS data structure, the specific women’s module used, and the variables selected for this study. We now provide greater detail on the questions informing each reproductive health indicator. These clarifications have been added to the Data Sources and Study Variables and Their Measurement.

Comment 3. The study variables and their measurement methods require further details.

Reply: Thank you for your thoughtful comment. We would like to note that the detailed definitions, coding schemes, and rationale for all the variables included in the analysis are presented in Table 1 of the manuscript. This table outlines how each component of the Reproductive Health Index (RHI) is derived from the NFHS-5 data, ensuring transparency and replicability. We have also reviewed the surrounding text to ensure it appropriately references and explains the variables where needed.

Comment 4. In Table 2, the authors report that 75% of respondents have ever experienced a stillbirth or miscarriage. This figure appears inaccurate, as the NFHS report indicates only 7.3% for miscarriage and 0.9% for stillbirth. The authors should verify these figures.

Reply: Thank you for this insightful comment. We have carefully reviewed this issue and would like to clarify that our estimate is based on a subsample of currently married women aged 15–49 who had at least one birth in the five years preceding the survey, rather than all women of reproductive age. This differs methodologically from the NFHS-5 national report, which calculates the prevalence of stillbirth and miscarriage across the entire sample of surveyed women. Furthermore, our operationalisation of the variable “ever experienced stillbirth/miscarriage” includes only stillbirth and miscarriage, and excludes abortion, in line with the focus of our Reproductive Health Index (RHI) on involuntary and health-related pregnancy losses. Including abortion, a decision that is often voluntary would not align with the conceptual objective of the index and might misrepresent the reproductive health burden the index is intended to capture. We have clearly noted this in the main text of the Methods section and a footnote has been included below Table 1 to reinforce this decision.

Comment 5. In Table 3, the mean RHI varies by women’s age, age at first marriage, and age at first birth. While RHI increases with higher age at first marriage and first birth, it appears to decrease with advancing age of women. This pattern needs further explanation from the authors to clarify the underlying reasons.

Reply: Thank you for pointing this out. We agree that this is an important pattern that requires clarification. We have now added a brief explanation in the revised results section. While women who marry or give birth at later ages tend to have better reproductive health, likely due to greater physical readiness and increased care-seeking, RHI tends to decline among older women. This could reflect the cumulative effects of reproductive strain, repeated childbearing, or reduced access to health services over time.
